# Scattering of Sculpted Light in Intact Brain Tissue, with implications for Optogenetics

**DOI:** 10.1038/srep11501

**Published:** 2015-06-25

**Authors:** Itia A. Favre-Bulle, Daryl Preece, Timo A. Nieminen, Lucy A. Heap, Ethan K. Scott, Halina Rubinsztein-Dunlop

**Affiliations:** 1 School of Mathematics and Physics, The University of Queensland, Brisbane QLD, Australia; 2 School of Biomedical Sciences, The University of Queensland, Brisbane, QLD, Australia; 3 Queensland Brain Institute, The University of Queensland, Brisbane, QLD, Australia

## Abstract

Optogenetics uses light to control and observe the activity of neurons, often using a focused laser beam. As brain tissue is a scattering medium, beams are distorted and spread with propagation through neural tissue, and the beam’s degradation has important implications in optogenetic experiments. To address this, we present an analysis of scattering and loss of intensity of focused laser beams at different depths within the brains of zebrafish larvae. Our experimental set-up uses a 488 nm laser and a spatial light modulator to focus a diffraction-limited spot of light within the brain. We use a combination of experimental measurements of back-scattered light in live larvae and computational modelling of the scattering to determine the spatial distribution of light. Modelling is performed using the Monte Carlo method, supported by generalised Lorenz–Mie theory in the single-scattering approximation. Scattering in areas rich in cell bodies is compared to that of regions of neuropil to identify the distinct and dramatic contributions that cell nuclei make to scattering. We demonstrate the feasibility of illuminating individual neurons, even in nucleus-rich areas, at depths beyond 100 μm using a spatial light modulator in combination with a standard laser and microscope optics.

Optogenetics, the use of proteins to manipulate or report on neural physiology using light, is an important emerging technology in the field of neuroscience. This is because optogenetic approaches allow control over genetically prescribed populations of neurons that may be distributed across wide areas of the brain^1^,^2^,^3^,^4^,^5^. In principle, this should permit the interrogation of functional circuits, including their behavioural relevance, with single cell resolution^6^,^7^. This potential is currently constrained by technical obstacles related to genetic targeting, protein engineering, and especially targeted illumination.

The optical properties of the brain have been the focus of extensive research^8^,^9^, much of it relating to the propagation of light through different thicknesses of neural tissue^10^,^11^,^12^,^13^,^14^. In the context of optogenetics, these properties are important because they factor in how powerful a light source must be, and at what distance from the targeted cell, to drive a physiological effect^5^,^15^,^16^. Most of these studies, like most optogenetic experiments reported to date, have used unsculpted light, often from optical fibers, to illuminate whole regions of tissue. In order to use optogenetics for cellular or microcircuit-level experiments, it is necessary to restrict illumination to small (ideally single cell) volumes. For this approach, the scattering properties of neural tissue on sculpted light are of paramount importance.

As referred to above, optimal circuit mapping requires the temporally-controlled illumination of one or more prescribed neuron-sized volumes distributed throughout a large and heterogeneous milieu of neural tissue. Traditional approaches for optogenetic illumination, such as the delivery of laser light through an optical fibre, are insufficient for this sort of experiment. Digital micromirror devices (DMDs) have been used to exert this level of spatial and temporal control over brain sections^17^,^18^, but due to their low efficiency when used as binary holograms and poor axial focusing when imaged into tissue, they are unsuitable for experiments at depth in intact brains. Spatial light modulators (SLMs) have the unique property of sculpting light in three dimensions^19^,^20^ and therefore can provide simultaneous cell-specific targeting in multiple planes^21^,^22^,^23^. One example, using an SLM and a calcium-sensitive protein to detect activity in intact zebrafish brains, has recently been reported^24^.

As such, SLMs are poised to become the tool of choice for optogenetic circuit analyses^25^,^26^ in small animal models, as well as in thick slice preparations from larger brains^27^,^28^. They will only be effective, however, if the effects of scattering through neural tissue are overcome so that the light’s focus and intensity are sufficient to drive physiological effects at depth^29^,^30^,^31^,^32^. In this study, we have carried out detailed analyses and modelling of the impacts of scattering on SLM-sculpted spots at different depths of neural tissue *in vivo*, including the differential scattering that takes place in brain regions where cell nuclei are abundant or sparse.

## Results

**Figure 1:**
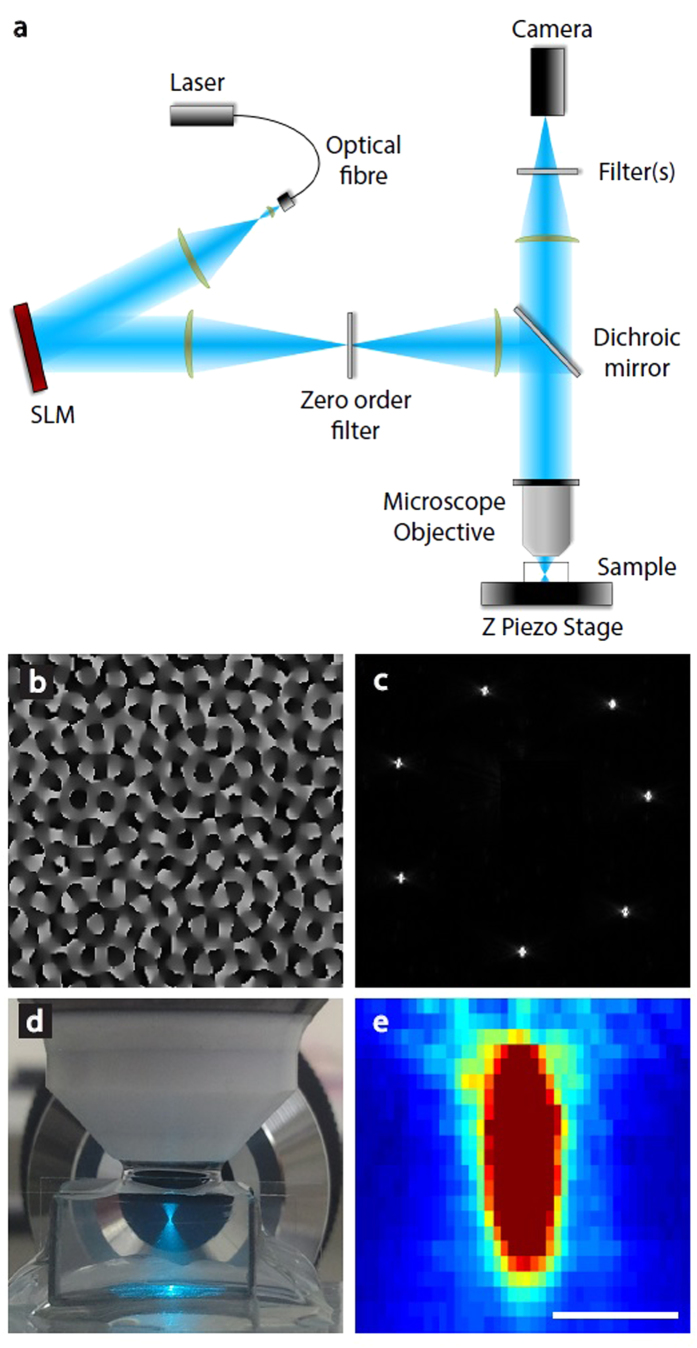
Light sculpting and detection. **a,** A beam expander collimates the light from a 488 nm laser onto an SLM. A telescopic configuration of lenses images the SLM pattern onto the back focal plane of the microscope objective. A filter is used to block zero order light from the SLM. **b,** An example of an SLM pattern used to generate seven spots (**c**). **d**, A sculpted beam focusing through agarose. **e**, Intensity plot of a diffraction limited spot generated on this microscope. Scale bar in **e** represents 2 μm.

Our microscope setup (Fig. 1) uses an SLM to sculpt light from a 488nm laser, targeting illumination to the focal plane of a 40x, 0.8 NA water immersed objective, and images backscattering using the same objective. Examples of an SLM pattern and its corresponding hologram are shown in Fig. 1b,c, respectively. Such spots are diffraction limited in size (Fig. 1e), with a full-width half-maximum (FWHM) of roughly 0.13 μm in X and Y and 0.3 μm in Z when using a 40X objective with an NA of 0.8.

**Figure 2:**
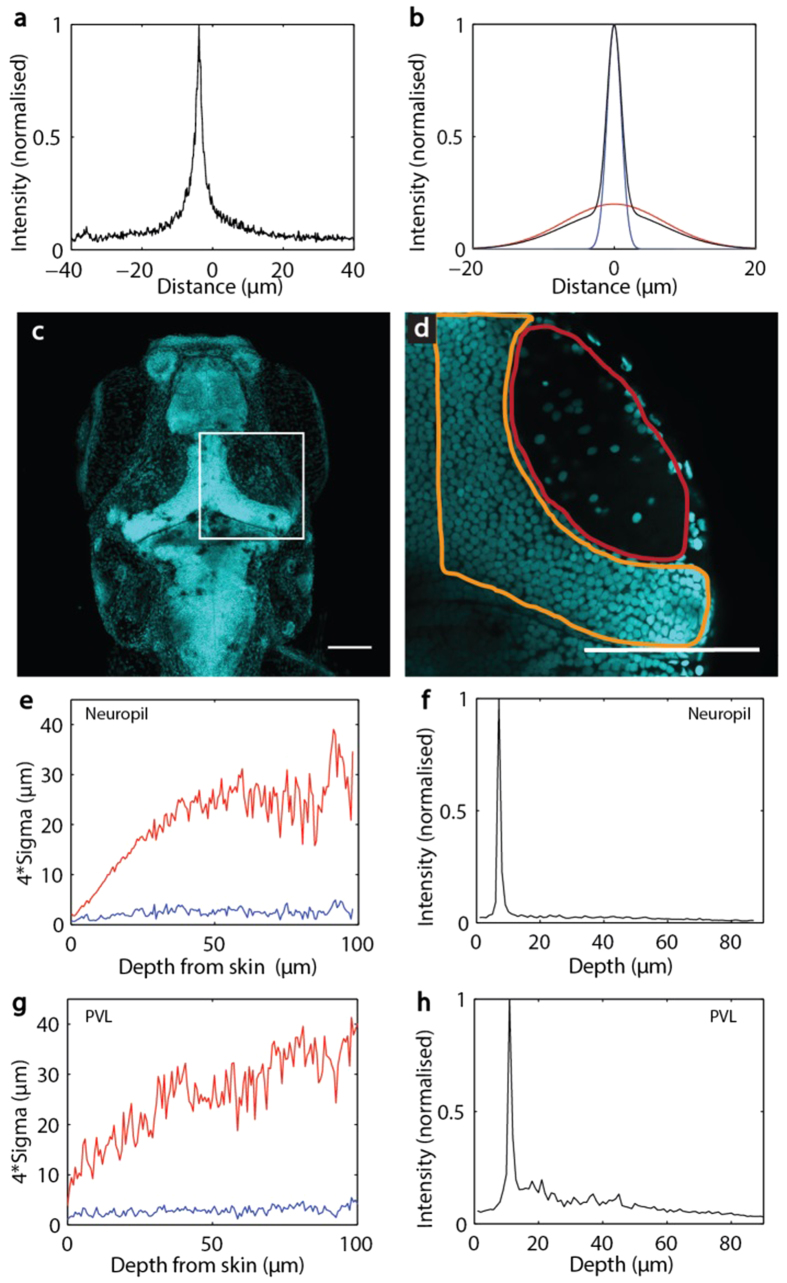
Backscattered intensity profile with depth in different types of neural tissue. **a**, Example of a backscattered intensity profile recorded from a spot focused 100 μm deep inside the optic tectum. **b**,This profile can be fitted with a sum of two Gaussians (red and blue with the sum shown in black). **c**, Maximum intensity projection of a confocal stack from a 6dpf larva, stained with DAPI. **d**, The boxed area in c is represented with the neuropil indicated by a red oval and the periventricular layer, PVL, bounded by orange. e-h, The evolution of the fitted Gaussian width with depth is shown for the tectal neuropil (e) and PVL (g), where the narrow and intense Gaussian is represented in blue, and the wider and weak Gaussian is represented in red. f and h show experimentally measured backscattering intensity maxima with depth in neuropil and PVL, respectively. Scale bars in c and d represent 100 μm.

To study the scattering of light in neural tissue, we immobilised 6 day post-fertilisation (dpf) zebrafish larvae in agarose, and focussed diffraction-limited spots at different depths within the brain. The backscattered intensity profile (Fig. 2) has a characteristic shape, with its width and intensity varying depending on depth of focus and region of the brain. The evolution of the backscattered intensity profile, in terms of its width and maximum intensity, can be parameterised by fitting the observed intensity profiles with a sum of two Gaussians (Fig. 2b).

The evolution of backscattered light as a function of depth depends on the density of cell nuclei in the tissue (Fig. 2c-h). Backscattering shows a peak corresponding to the larva’s skin (main peak in Fig. 2f,h), which reflects heavily due to the mismatch of its refractive index with that of agarose. As the focal spot is moved deeper, the effects of the skin decrease, and we can observe the effect of scattering within the brain. In the tectal neuropil, a region nearly devoid of nuclei (red oval in Fig. 2d, see Fig. S1), the width of the wide Gaussian increases with depth (Fig. 2e), and without peaks in backscattering across depth (Fig. 2f). In the PVL, which is rich with nuclei (bounded by orange in Fig. 2d, see Fig. S1), the wider Gaussian’s width increases constantly (Fig. 2g), with individual nuclei causing peaks of backscattering at various depths (Fig. 2h). This presumably results from mismatches in the refractive indices for nuclei, which contain nucleic acids, and the surrounding milieu of principally composed of membrane, fluid, and protein. The evolution of the widths of the two Gaussians with depth is also presented in Fig. 2. Interestingly, the main Gaussian (blue, Fig. 2e,g) does not widen significantly while the secondary Gaussian does (red, Fig. 2e,2g). This implies that the combined scattering from these Gaussians will still allow relatively intense focal illumination at depth.

The intensity profiles shown in Fig. 2 represent backscattered light, which does not necessarily match the spatial distribution of light within the brain. Gauging the actual distribution is critical for optogenetic manipulations, but it is very difficult to observe this light within an intact specimen. To address this, we have built a theoretical model, using the Monte Carlo method, which will allow us to infer the actual structure of the light within the brain based on observations of backscattering.

We have modelled brain tissue as a homogeneous background medium with discrete scatterers. The light is modelled as a collection of photons, each of which has a given probability per unit distance of encountering a scatterer. The density of scatterers can be varied to simulate different types of neural tissue such as the tectal neuropil and PVL. The scattering properties of each scatterer are represented by the scattering cross-section and anisotropy parameter^33^,^34^, which can be calculated using Lorenz–Mie theory^35^,^36^ for roughly spherical scatterers such as nuclei.

In neural tissue, scattering bodies will include nuclei and other organelles as well as the interfaces between membranes and extracellular space^12^,^37^. As nuclei are relatively large, they scatter predominantly at low angles in the forward direction, and are therefore expected to impact the distribution of light near the focal point. In our model, we have ignored the effects of smaller scatterers, which will tend to scatter more isotropically, distributing light throughout the medium, and affecting the region around the focal spot minimally. Similarly, we have ignored the effects of skin reflection. The overall reflectivity of the skin is small, and the effect on light distribution within the brain is a uniform reduction in energy density.

**Figure 3:**
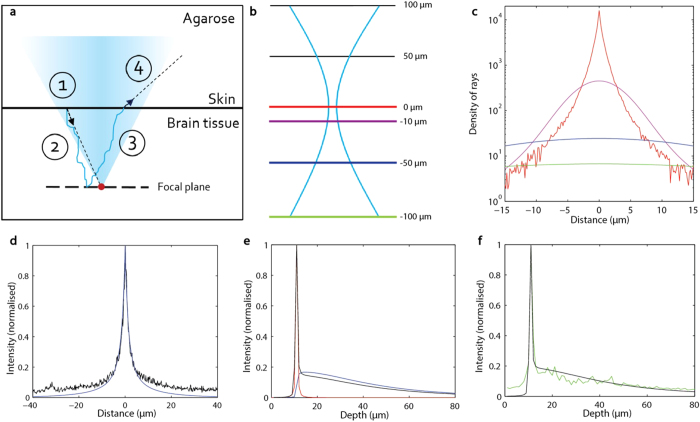
Monte Carlo simulation of backscattering. **a**, Different stages of scattering (the contributions from agarose and skin are ignored): 1. Gaussian distribution of 4 × 106 photons on the skin. 2. Trajectory calculation in brain tissue for each photon. 3. Backscattered trajectory calculation. 4. Recording of each photon’s final position and direction exiting the brain. **b**, A schematic representation of beam shape through 200 μm of tissue, with the focal point at 100μm depth. **c**, Calculated contributions of intensity and from depths indicated in b. **d**, Sum of all calculated contributions from 201 planes (blue) registered against experimental observations (black). **e**, Calculated backscattering from skin (red) and brain (blue), and total calculated backscattering (black), by depth. **f**, total calculated backscattering (black, drawn from e) registered against experimental measurements in the PVL (green, drawn from Fig. 2h).

For these reasons, only scattering by nuclei was included in our model. In order to identify appropriate parameters for nuclear size and density, we imaged nuclei in the tecta of fixed larvae using DAPI staining and confocal microscopy (see Supplementary Methods). Refractive index values for nuclei were based on published measurements^38^,^39^, which were then refined empirically within our model to deliver a value of 1.35, versus 1.34 for surrounding neural tissue (see Supplementary Information). An overview of the model, including each step that is calculated using the parameters described above, is shown in Fig 3a.

In Fig. 3b, we show the calculation of intensity contributions from different planes to the light detected by the camera. Of 4 × 10^6^ photons included in this simulation, the focal plane (red, Fig. 3b,c) contributes more than 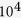 backscattered photons to the center of the focal spot and very few at distances greater than 10 μm from the focal point, while a plane 100 μm deeper than the focal plane (green, Fig. 3b,c) contributes a much weaker and flatter profile. Across these depths, the focal plane contributes mostly to the peak and distant planes contribute most strongly to the offset, while planes near but not at the focal plane contribute the wings of the intensity profile. The resulting sum of all contributions (201 planes, at 1μm intervals, from 100 to −100 μm depth relative to focus) is shown in Fig. 3d (blue line). This simulation matches well our experimental measurements (black line, Fig. 3d, drawn from Fig. 2a). The main difference is in the offset, which is greater in the experimental data than in the simulation, due to the effects of skin and small scatterers not accounted for in our model. So, while the experimentally observed backscattering intensity profile could be fitted, to a first approximation, to a sum of two Gaussians (Fig. 2a,b), the results above show that the actual profile is the sum of a theoretically infinite number of Gaussians, each deformed to a different degree by scattering.

We can also calculate the decrease in the maximum intensity of the backscattered light with depth (see Methods), shown in Fig. 3e. Reflection from the skin is incorporated using a point spread function for out-of-focus planes as above. Combining the contributions from the skin (Fig. 3e, red) and the brain (Fig. 3e, blue) produces a model of backscattering intensity versus depth (Fig. 3e,f, black) that agrees with experimental results from the intact brain (Fig. 3f, green, drawn from Fig. 2h). The sawtooth nature of the experimental curve is a product of nuclei, which serve as points of high backscattering. In contrast, the model produces a smooth curve, since scattering is simulated at the level of individual photons in a homogeneous medium, and is therefore not punctuated spatially.

**Figure 4:**
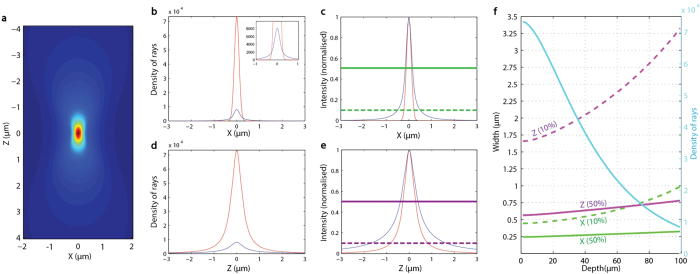
Intensity profile calculated inside brain tissue with Monte Carlo method. **a**, 2D (X, Z) representation of voxel size with scattering. b-e, Comparison of intensity profile with (blue curve) and without scattering (red curve) along X and Z. c and e show normalised intensity in b and d respectively. From these normalised figures and the power of the laser, the volume illuminated to a given intensity can be deduced. f, Evolution of spot width (with 10% or 50% maximum intensity threshold) inside brain tissue on the X and Z axes with depth.

The match between the calculated and observed backscattering profiles and their evolution with depth suggests that our calculated distribution of light is essentially correct. This permits us to calculate the intensity profile near the focal spot, in three dimensions (Fig. 4a). The transverse and axial widths of the focal spot are of great interest, since these determine the lower spatial bounds of optogenetic manipulations that can be delivered.

In the transverse direction (X and Y, Fig. 4b,c), scattering spreads the light (inset, Fig. 4b). In the axial direction (Z, Fig. 4d,e), the beam is not spread but the maximum intensity is reduced by the transverse spreading of the focal spot. As a result, the focal width at half maximum (FWHM) in the axial direction is increased (magenta line, Fig. 4e). In the absence of scattering, the size of the focal volume is 0.13 μm (transverse) by 0.29 μm (axial), increasing to 0.16 μm by 0.39 μm at a depth of 100 μm depth.

Using calculations like those in Fig. 4c,e, carried out at every depth, we have deduced the volume of illumination as a proportion of laser power. In Fig. 4f we show the evolution of the volume of illumination in both X and Z with two different thresholds: 10% focal peak intensity, and 50% focal peak intensity. At all depths, the volume illuminated to 50% peak intensity remains relatively small, reaching 0.32 μm and 0.78 μm for X and Z, respectively, at 100 μm depth. The volume illuminated to 10% peak intensity is markedly larger (0.98 μm and 3.35 μm for X and Z, respectively, at the same depth). With the exception of the Z-axis for the 10% peak intensity volume, these measurements are within the bounds of a typical neuronal cell body (diameters of 4.84, 5.68, and 2.6 μm in X and Y and Z-axes, respectively, for the tectal neurons in this study, see Figure S2), suggesting that single-cell illumination should be feasible to at least 100μm depth of neural tissue.

## Discussion

These results demonstrate that, through the use of an SLM, volumes equivalent to single neurons can readily be illuminated at depth in intact neural tissue. Importantly, we show this to be true under conditions that are not optimal for such focusing. For instance, we have generated our estimates in a nucleus-dense part of the nervous system, and have used a 488nm laser. Performance should only improve in clearer tissue, or with the use of longer wavelength lasers used for 2-photon excitation. We also measured the sizes of cell nuclei, which will inevitably be smaller than the cell bodies themselves.

The ability to restrict illumination to single targeted neurons, or to numerous selected neurons in densely packed tissue, provides a potentially powerful tool for circuit analysis. This capability should allow the contributions of individual neurons to local circuit dynamics to be gauged. This would simply involve driving activity in a targeted cell, while observing calcium or voltage dynamics in cells potentially connected to it. Using the same approach, the circuit impacts of silencing a specific neuron (with an optogenetic silencing protein), could be observed. Since SLMs can be used to project numerous points of light in three dimensions, they could be used to drive temporally-controlled patterns of activity throughout a small brain. This would open the door to testing such patterns, at single neuron resolution, for their ability to drive downstream circuit responses, regulate neural plasticity, or elicit behavior.

The results reported here also have design implications for future optogenetic experiments. Since the dramatic broadening of the focal spot at depth occurs at low intensity (Fig. 4), experiments should aim for a balance of power (determined by the light source) and sensitivity (determined by actuator efficiency, expression level, and the target cell’s physiological properties) that leads to physiological manipulations only when a large portion of the beam’s maximum intensity is brought to bear on the target cell. This, in turn, may relieve the need for very powerful illumination (which may cause phototoxicity and behavioural confounds), more sensitive actuators, and high expression levels of actuators (which could have adverse effects on the cells). These results, however, highlight the risk of triggering off-target manipulations in adjacent cells, particularly in the Z-axis, if this balance is tipped too sensitively.

## Methods

### Microscopy and light sculpting

For illumination, we used a 150 mW 488 nm laser (OBIS) coupled to an optical fiber. The laser light was expanded and collimated to fill the reflective surface of a PLUTO VIS Holoeye Spatial Light Modulator (SLM). The pattern created by the SLM was projected on the back focal plane of a 40x water immersion microscope objective (Olympus LUMPLFLN 40XW, 0.8 NA), slightly overfilling it. The total efficiency of the SLM for single spot generation was about 60%, because some of the light reflects into the zero order rather than being directed to the desired spot position. In order to exclude the non-diffracted light, we used a spatial filter in the optical path that cuts out the zero order light. This holographic beam pattern was coupled to the microscope, providing illumination with desired geometry and focal plane within the brain of the zebrafish larva.

Images were acquired by collecting the reflected light through the same microscope objective that was used for the illumination. The reflected light passed through a dichroic mirror and was directed to a CMOS camera (PCO Edge) after passing through a single lens and filters.

### Sample preparation, imaging, and nuclear measurements

All experiments were carried out under approval of the University of Queensland Animal Welfare Unit (approval SBMS/305/13/ARC). Larvae were raised at 28.5 °C. At 6dpf, they were fixed overnight at 4 °C in 4% paraformaldehyde in PBS (pH 7.5). Larvae were then washed twice in PBS and transferred for cryopreservation into 30% sucrose and 0.02% sodium azide in PBS. Prior to whole mount immunohistochemistry, the lower jaw, swim bladder, gut, and tail of larvae were removed to provide better penetration to the tectum. Animals were stained with the nuclear stain 4’,6-diamidino-2-phenylindole (DAPI, Life Technologies) overnight at 4 °C at a concentration of 1:1000, and washed three times for five minutes each in PBS. Fixed and stained animals were mounted in 1.5% low melting temperature agarose (Progen Biosciences) in standard E3 media, and were imaged on a Zeiss 710 confocal microscope.

Confocal stacks of DAPI-stained brains were taken at 0.2 μm intervals. The percentage of the volume occupied by cell nuclei in PVL and neuropil was estimated by thresholding images in ImageJ (version1.49d) software (United States National Institutes of Health) and measuring the percentage of the area of that slice occupied by thresholded pixels. Nuclear dimensions were judged by measuring the maximal medial-lateral (X-axis) and rostral-caudal (Y-axis) diameters in ImageJ, or by multiplying the number of slices in which each nucleus appears by 0.2 μm (Z-axis).

### Monte Carlo method

The aim of the Monte Carlo calculation is to determine the trajectories of the rays entering the brain tissue and record their positions and directions in each plane of interest. The different stages of scattering are shown in Fig. 3a and represent the main steps in the Monte Carlo calculation. The details of considerations and approximations for these calculations are presented in Supplementary Information.

As we were using a Gaussian beam focusing at a certain depth, we first assumed a Gaussian distribution of rays leaving the skin in the direction of the focal spot. Next, the path of each ray, including the effect of scattering, was calculated using Monte Carlo method. Once the ray reached the plane of interest, its position and direction were recorded. With the calculation of a sufficient number of rays (depending on the resolution), we were able to determine the distribution of light within the brain tissue in the plane of interest.

For the calculation of backscattered light, additional steps were required. Once a ray reaches the plane of interest, it has a certain probability of being transmitted or refracted. With some judgements made on the scattering phase function (see Supplementary Information), we assumed that every ray was reflected, and we calculated the path of the ray on the way back to the skin with the Monte Carlo method. Finally, we recorded their positions and directions and reconstructed the image expected on the camera (see Supplementary Information). In order to adequately take diffraction into account in our ray optics simulation we convolved the Gaussian beam waist of an ideal beam with the result of the Monte Carlo simulation.

## Additional Information

**How to cite this article**: Favre-Bulle, I. A. *et al*. Scattering of Sculpted Light in Intact Brain Tissue, with implications for Optogenetics. *Sci. Rep*. **5**, 11501; doi: 10.1038/srep11501 (2015).
