# Publisher Correction: Scattering of Sculpted Light in Intact Brain Tissue, with implications for Optogenetics

**DOI:** 10.1038/s41598-025-92455-1

**Published:** 2025-03-14

**Authors:** Itia A. Favre-Bulle, Daryl Preece, Timo A. Nieminen, Lucy A. Heap, Ethan K. Scott, Halina Rubinsztein-Dunlop

**Affiliations:** 1 School of Mathematics and Physics, The University of Queensland, Brisbane, QLD Australia; 2 School of Biomedical Sciences, The University of Queensland, Brisbane, QLD Australia; 3 Queensland Brain Institute, The University of Queensland, Brisbane, QLD Australia

Correction to: *Scientific Reports* 10.1038/srep11501, published online 25 June 2015

The Supplementary Information file was omitted from the original version of this Article. The Supplementary Information is now linked to this correction notice.

## Supplementary Information

Supplementary Information.

