# Supplementary material for: Publisher Correction: Scattering of Sculpted Light in Intact Brain Tissue, with implications for Optogenetics
Source: Sci Rep. 2025 Mar 14;15:8866. doi: 10.1038/s41598-025-92455-1 (PMC11909273; doi:10.1038/s41598-025-92455-1)
Supplement: Supplementary file 1 — Supplementary Information. [file 41598_2025_92455_MOESM1_ESM.pdf]

Scattering of Sculpted Light in Intact Brain Tissue, with implications for Optogenetics

Itia FAVRE-BULLE[1], Daryl PREECE[1], Timo A. NIEMINEN[1], Lucy A. HEAP[2], Ethan K. SCOTT[2,3*] and Halina RUBINSZTEIN-DUNLOP[1*]

[1]*School of Mathematics and Physics, The University of Queensland, Brisbane QLD, Australia*

[2]*School of Biomedical Sciences, The University of Queensland, Brisbane, QLD, Australia*

[3]*Queensland Brain Institute, The University of Queensland, Brisbane, QLD, Australia*

* Corresponding authors: Halina Rubinsztein-Dunlop (halina@physics.uq.edu.au) and Ethan K. Scott (ethan.scott@uq.edu.au)

This supplement has two parts. In part A, we provide measurements of cell density and nuclei sizes in the periventricular layer region of Zebrafish brain. In part B, we provide all equations and assumptions made for our Monte Carlo model.

A. **DAPI staining**

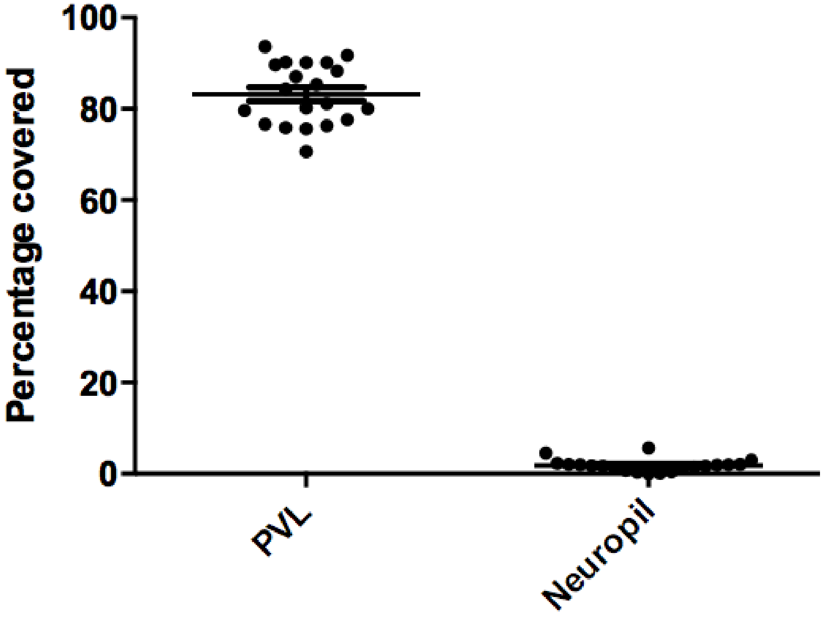

Figure S1: Proportion of neuropil and PVL occupied by nuclei. The percentage of neuropil area occupied by nuclei in the neuropil versus the periventricular layer (PVL). Points represent the percentage occupied in individual confocal slices (20 slices across two larvae).

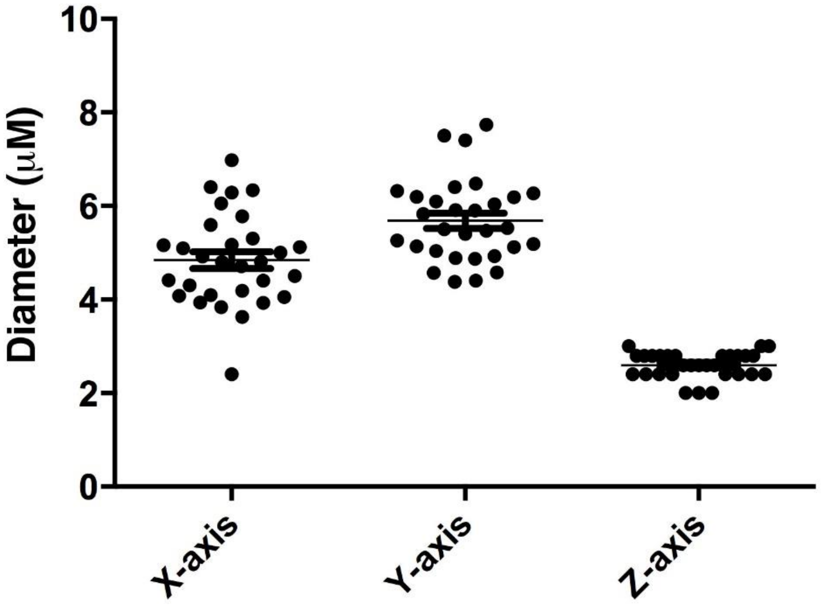

Figure S2: Quantification of nucleus size. The diameter of periventricular layer nuclei for the X (medial-lateral), Y (rostral-caudal), and Z (dorsal-ventral) axes. Points represent individual nuclei (30 nuclei across two larvae).

B. Monte Carlo method

The Monte Carlo method provides a fast and simple method of simulating scattering in a scattering medium composed of randomly distributed discrete scattering particles embedding in a background medium[1,2]. The probability per unit length P of a ray being scattered is

$$P = CN$$

where C is the scattering cross-section of the scattering particles and N is their number density. Therefore, the distribution of distances Δd travelled by rays between scattering events is

$$\Delta d = \frac{-\ln(R)}{C}$$

Where R is a function giving a random value between 0 and 1. For a spherical particle, Lorenz–Mie theory provides an analytical solution for the scattering of a plane wave[3,4]. First, the Mie coefficients a_n and b_n are given by

$$a_n = \frac{\left[\frac{D_n(mx)}{m} + \frac{n}{x}\right]\psi_n(x) - \psi_{n-1}(x)}{\left[\frac{D_n(mx)}{m} + \frac{n}{x}\right]\xi_n(x) - \xi_{n-1}(x)}$$

$$b_n = \frac{\left[\frac{mD_n(mx)}{m} + \frac{n}{x}\right]\psi_n(x) - \psi_{n-1}(x)}{\left[\frac{mD_n(mx)}{m} + \frac{n}{x}\right]\xi_n(x) - \xi_{n-1}(x)}$$

where ψ_n and ξ_n are the Ricatti–Bessel functions and the logarithm derivative[5,6]

$$D_n(\rho) = \frac{d}{d\rho}\ln\psi_n(\rho)$$

m is the complex refractive index of the sphere relative to the medium. $x = ka$ is the size parameter, where a is the radius of the sphere and k the wavenumber of incident light. The scattering cross section is then given by[5,6]

$$C = \frac{2\pi}{k^2}\sum_{n=1}^{\infty}(2n+1)[|a_n|^2 + |b_n|^2]$$

As we are only considering scattering by non-absorbing particles, the absorption cross section is zero, and the scattering and extinction cross sections are equal. Note that using this method, each scattering event takes place at a different location. The scattering particles do not have particular positions within the medium, which is treated as a homogeneous isotropic scattering medium characterised by the probability of scattering per unit length, or equivalently, the mean free path.

When a ray is scattered, it will be scattered in a random direction. The distribution of scattering directions is not uniform; for the case of low-contrast scatterers of moderate or large size such as we consider here, the scattering is predominantly in the forward direction, with only very weak

backscattering. In principle, we could calculate the distribution of scattering angles (θ, φ) exactly, using Lorenz–Mie theory, but it is convenient to assume that the distribution is

$$\theta = \cos^{-1}\left(\frac{1}{2g}\left[1 + g^2 - \left(\frac{1-g^2}{1_g+2gR}\right)^2\right]\right),$$

$$\varphi = 2\pi R.$$

Where g is the anisotropy parameter, which is given from the Mie coefficients by[6]

$$g = \frac{4\pi}{k^2} C \sum_n \frac{n(n+2)}{n+1}\left[\mathrm{Re}(a_n a_{n+1}^* + b_n b_{n+1}^*) + \frac{2n+1}{n(n+1)}\mathrm{Re}(a_n b_n^*)\right]$$

The propagation of each ray can then be simulated in the region of interest, until the ray reaches the edge of the region. If we were considering absorbing particles or an absorbing medium, each ray would also have a probability of being absorbed per unit length. Once the paths of a large enough number of rays have been simulated, the density of rays provides an approximation of the irradiance in the scattering medium.

Since we are calculating the scattering of a focussed Gaussian laser beam, we begin with an initial distribution of rays given by

$$X_0 = N_r\left(\frac{Z_{focaldepth} \cdot \tan\left(\sin^{-1}\frac{NA}{n}\right)}{3}\right),$$

$$Y_0 = N_r\left(\frac{Z_{focaldepth} \cdot \tan\left(\sin^{-1}\frac{NA}{n}\right)}{3}\right),$$

$$Z_0 = 0.$$

Where N_r is a function giving a random number chosen from a normal distribution with mean of 0 and variance equal to the argument in the brackets. NA is the numerical aperture of the microscope objective and n the refractive index of water. Noting that in the absence of scattering, this gives the unphysical result of a focal spot of zero size and infinite irradiance, we can correct for this by calculating the convolution of the Monte Carlo irradiance in a plane with the focal spot of the unscattered beam, calculated using wave theory. For the numerical aperture considered here, the paraxial formula is sufficiently accurate[7], giving a Gaussian focal spot

$$I(x,y,z) = \left(\frac{w_0}{w(z)}\right)^2 e^{-2\left(\frac{x^2+y^2}{w(z)^2}\right)}$$

Where w_0 is the beam waist defined as

$$w_0 = \frac{\lambda}{\pi . NA}$$

And

$$w(z) = w_0\sqrt{1 + \left(\frac{z}{z_r}\right)^2}$$

$$z_r = \frac{\pi w_0^2}{\lambda}$$

After this convolution is calculated, the distribution of light within the brain tissue has been found. The next step is to find the backscattered image. Since the backscattering is weak, it is impractical to trace rays until enough of them exit the region of interest in the backward direction. Instead, once the light distribution in the brain tissue has been found, we assume that the backscattering is proportional to the irradiance, and generate a set of backscattered rays, which we track using the Monte Carlo method, until they exit the region of interest. Since these rays originate from different planes, each a different distance from the focal plane of the objective lens used to image the backscattered light, the light from most planes will be out of focus; only light from the focal plane itself will be in focus. In addition, the resolution of the lens is limited even in its focal plane. If we consider an unscattered Gaussian beam focussed by the lens, the cross section of this beam in any given plane is the point spread function of the lens in that plane. Therefore, we can take the convolution of the light from each plane with this point spread function to determine the contribution of that plane to the backscattered image.

Since the beam is rotationally symmetric, and the medium is homogeneous and isotropic, the scattering is, on average, rotationally symmetric about the beam axis, and we can average the results in different planes about the beam axis to obtain a more accurate average ray density.

References

1. Patterson, M.S, Wilson, B.C. & Wyman, D.R. The propagation of optical radiation in tissue. I. Models of radiation transport and their application. *Lasers in Medical Science,* **6**, 155-168 (1991).
2. Flock, S.T., Patterson, M.S., Wilson, B.C. & Wyman, D.R., Monte Carlo modeling of light propagation in highly scattering tissues. I. Model predictions and comparison with diffusion theory. *IEEE Transactions on Biomedical Engineering*, **36**, 1162-1168 (1989).
3. Lorenz, L., Lysbevægelsen i og uden for en af plane Lysbølger belyst Kugle. *Videnskabernes Selskabs Skrifter*, **6**, 2-62 (1890).
4. Mie, G., Beiträge zur Optik trüber Medien, speziell kolloidaler Metallösungen. *Annalen der Physik,* **25**, 377-445 (1908).
5. Van de Hulst, H.C., Light scattering by small particles, Wiley, New York (1957).
6. Bohren, C.F. & Huffman, D.R., Absorption and Scattering of Light by Small Particles, pp. 6.1-6.21 in Bass, M. et al. (eds), Handbook of Optics Vol. I, McGraw-Hill, New York (1998).
7. Nieminen, T.A., Rubinsztein-Dunlop, H. & Heckenberg, N.R., Multipole expansion of strongly focussed laser beams. *Journal of Quantitative Spectroscopy and Radiative Transfer*, 79-80, 1005-1017 (2003).
